# Individual- and Neighborhood-Level Factors of Measles Vaccination Coverage in Niamey, Niger: A Multilevel Analysis

**DOI:** 10.3390/vaccines10091513

**Published:** 2022-09-10

**Authors:** Mika Kondo Kunieda, Mahamane Laouali Manzo, S. V. Subramanian, Masamine Jimba

**Affiliations:** 1 Department of Community and Global Health, The University of Tokyo Graduate School of Medicine, Tokyo 113-0033, Japan; 2 Takemi Program in International Health, Global Health and Population Department, Harvard T.H. Chan School of Public Health, Boston, MA 02115, USA; 3 Faculty of Policy Management, Keio University, Kanagawa 252-0882, Japan; 4 Ministry of Public Health, Niamey BP 613, Niger; 5 Harvard Center for Population and Development Studies, Cambridge, MA 02138, USA; 6 Department of Social and Behavioral Sciences, Harvard T.H. Chan School of Public Health, Boston, MA 02115, USA

**Keywords:** measles, vaccination coverage, individual-level factors, neighborhood-level factors, multilevel logistic model, Niger, Western Africa

## Abstract

Vaccination is a proven equitable intervention if people take advantage of the opportunity to get vaccinated. Niger is a low-income country in West Africa, with a 76% measles 1 vaccination coverage rate in 2016. This study was conducted to identify individual- and neighborhood-level factors that could improve measles 1 vaccination coverage in Niamey, the capital. In October 2016, 460 mothers with children aged 12–23 months were surveyed. The outcome was to determine whether the mother’s child had been vaccinated against measles 1 or not. For individual-level variables of measles 1 vaccination status, the following were included: mother’s age group, mother tongue, maternal education level, husband’s job, where the mother gave birth (at home or at a health center) and whether the mother discussed vaccination with friends. Neighborhood-level factors were access time to the health center, household access to electricity, and a grand-mean-centered wealth score. Multilevel logistic regression analysis was performed. At the individual-level, primary and secondary-educated mothers were more likely to vaccinate their children against measles 1 (aOR 1.97, 95% CI 1.11–3.51). At the neighborhood-level, no factors were identified. Therefore, a strengthened focus on equity-based, individual factors is recommended, including individual motivation, prompts and ability to access vaccination services.

## 1. Introduction

In 2015, the world rallied around the Sustainable Development Goals (SDGs) for the well-being of all [1]. Vaccination interventions have been viewed as a means to rectify health inequalities, but studies have continued to describe inequalities in vaccination coverage [2]. Research has focused on determinants of full vaccination coverage [3,4,5,6,7], barriers [8] and vaccination acceptance [5,9]. These studies concluded that education and socio-economic status are critical to achieve the “no one left behind” goal. One study conducted in urban Niger long before the SDGs were implemented found that mothers’ education had a larger influence on full vaccination status than either household structure or economic status [7]. A recent study identified high birth order, high number of under-five children in the house, household wealth, lack of maternal education, lack of media access, and living in poorer neighborhoods as risk factors for missing vaccination opportunities [10]. Another meta-analysis of 12 East African Demographic Health Survey (DHS) studies identified mothers’ age, education, husband’s education, media exposure, birth interval from last child, number of antenatal care visits, post-natal care visits, place of delivery, child size at birth, number of children, wealth index, country, and community poverty as determinants of complete vaccination [11].

In recent years, studies have increasingly focused on identifying effective interventions to improve vaccination. A 2016 Cochrane Review identified (1) providing parents and other community members with vaccination information, (2) health education at facilities in combination with redesigned vaccination cards, (3) regular vaccination outreach, and (4) integration of vaccination with other health services as effective interventions to improve vaccination coverage in low-and middle-income countries [12]. An article by the 2019 Nobel Economics Prize winners identified the most effective tool to increase vaccination coverage as a combination of information hubs, SMS reminders, and progressive incentives for immunization. As a result of this unique combination, vaccination increased by 44% compared with the status quo [13].

Nonetheless, the poorest and least educated populations in low-income countries remain at higher risk for measles. The poorest populations face a higher risk of death from measles, owing to higher undernutrition rates and, consequently, a weaker immune system [14]. Vaccination services alone do not reduce this risk. Furthermore, the poorest populations also lack resources to access water and sanitation facilities, as well as preventive and timely curative care [15]. The urban poor live in densely populated, unaired, closed, close-contact quarters, which puts them at a higher risk of measles [16]. Contextual factors, such as urban environment, population density, and migration, have played a role in measles outbreaks [17].

Outbreak prevention factors include enabling poor mothers to vaccinate their children [18]. One study in Pakistan identified family discussions on vaccination as strongly associated with measles vaccination [19]. An Indian study used multilevel analysis and found that individual socioeconomic characteristics were a strong factor for non-vaccination [20]. Another multilevel study found that neighborhood characteristics influence individual behaviors to such an extent that they cannot be ignored [21]. Such a multilevel context is also found on the African continent and several multilevel model studies have been conducted on this multilevel African context [22,23,24,25]. However, none of these studies focused on measles alone, and the only Niger dataset analyzed was from 2006, as one of the twenty-four countries studied [25].

Niger is a low-income country [26], with an estimated average vaccination coverage rate of 76% for measles 1 in the 2016 WHO-UNICEF estimate [27]. Since measles vaccination coverage is under the optimal 95%, the risk of disease outbreaks remains. To date, only two studies have been published on the individual-level factors associated with full vaccination in Niger [6,7]. There is little evidence on how communities could encourage mothers to vaccinate their children against measles. This study was conducted to identify individual- and neighborhood-level factors that could improve measles 1 vaccination coverage in Niamey, Niger.

## 2. Methods

### 2.1. Study Area

Niger is a landlocked desert country in West Africa, with an estimated population of 16 million people in 2015 [28]. The capital Niamey’s population is estimated to be just over one million. The average annual population growth rate was 4.0% per year from 2010 to 2015, whereas the average annual urban population growth rate was 5.1% per year from 2010 to 2015 [29]. The five health districts in Niamey have population densities ranging from 824 to 4845 people/km^2^ [28]. The districts are further divided into neighborhoods, which are considered as community-level administrative units in this study. A recent map of land use and the distribution of concrete buildings and steel-sheet roofs demonstrates Niamey’s spatial expansion [30].

According to the 2012 DHS, nearly twenty-five percent of households in Niamey had an indoor water faucet; one-fourth of households had a water faucet within the concession, while approximately half of the households had to use a public water tap. A third of the surveyed households had an indoor improved toilet, half shared toilets with other families, and six percent had no access to sanitation facilities. The average household size in Niamey was 5.8 persons, while 17% of households had more than 9 persons. A quarter of the households had only one room, whereas forty-five percent of households had two rooms [31].

### 2.2. Study Design and Data Collection

The overall study design, sample size calculation, study participants, data sources, and study management are detailed in the work of Kondo Kunieda et al. [6]. In brief, a cross-sectional household survey was conducted in the capital of Niger, Niamey. Data on the full vaccination coverage and socioeconomic household characteristics of 460 children aged 12–23 months were collected. Of 445 children, 38% were fully vaccinated. Mothers who were satisfied with their health workers’ attitude and had correct vaccination calendar knowledge were more likely to have fully vaccinated children. Mothers who had completed secondary school were also associated with having fully vaccinated their children.

For the outcome of this study, measles 1 vaccination status was determined by the dates recorded in the MCH handbook. However, when a mother was unable to show her child’s MCH handbook, she was asked questions on household characteristics, knowledge, attitude, and actions related to child vaccination. When a mother showed her child’s MCH handbook, the surveyors copied all the dates of vaccination onto the survey questionnaire, photographed the page, and asked the mother the same questions as described above. 

All the data were entered and cleaned with Microsoft Excel and then exported to MLwiN 2.26 (Centre for Multilevel Modelling, University of Bristol, Bristol, UK) for preliminary statistical analyses. The final analyses presented in this paper were performed using Stata 16.1 for Windows (StataCorp LLC, College Station, Texas, USA). Analytical results were then visualized using QGIS Desktop 2.18.13 software.

### 2.3. Data Analysis

For individual-level variables of measles 1 vaccination status, the following were included: mother’s age group, mother tongue, maternal education level, husband’s job, where the mother gave birth (at home or at a health center) and whether the mother discussed vaccination with friends [13,19]. Neighborhood-level variables included a categorical variable for access time to the health center [5,32], a binary variable for household access to electricity [33], and a grand mean-centered wealth score [33].

Multilevel modeling was employed to assess the relevance of individual- and neighborhood-level factors in predicting measles 1 vaccination status [33,34]. Multilevel modeling also deconstructed the variance attributed to mothers or neighborhoods. The following five models were fitted: model 0 was a null model with no exposure variable, model 1 contained only individual-level variables, and model 2 contained only neighborhood-level variables. Model 3 was a multilevel model that contained all the individual- and neighborhood-level variables. The two-level regression model 3 is as follows:

$$Cov_{ij}∼\;N\left(XB,\Omega \right)$$

$$\begin{array}{cc} Cov_{ij}=\beta_{0ij}cons & +\beta_{1}no\;education_{ij}+\beta_{2}basic\;literacy_{ij}\\ & +\beta_{3}prim\;and\sec education{\;}_{ij}+\beta_{4}\sec plus\;education{\;}_{ij}\\ & +\beta_{5}mother\;15-19{\;yrs\;old}_{ij}+\beta_{6}mother\;20-24\;yrs\;old_{ij}\\ & +\beta_{7}mother\;25-29\;yrs\;old_{ij}+\beta_{8}mother\;30-34\;yrs\;old_{ij}\\ & +\beta_{9}mother\;35-39\;yrs\;old_{ij}\\ & +\beta_{10}mother\;40\;yrs\;old\;or\;more_{ij}+\beta_{11}Hausa_{ij}\\ & +\beta_{12}Zarma_{ij}+\beta_{13}Fulani_{ij}+\beta_{14}Tamachek_{ij}\\ & +\beta_{15}other\;{\left(languages\right)}_{ij}+\beta_{16}husband\;unemployed_{ij}\\ & +\beta_{17}husband\;informal\;worker_{ij}\\ & +\beta_{18}\;husband\;formal\;employment_{ij}\\ & +\beta_{19}\;husband\;public\;servant_{ij}+\beta_{20\;}gave\;birth\;at\;home_{ij}\\ & +\beta_{21}gave\;birth\;at\;health\;center_{ij}\\ & +\beta_{22}no\;discussion\;on\;vaccination\;with\;friends_{ij}\\ & +\beta_{23}discussed\;vaccination\;with\;friends_{ij}\\ & +\beta_{24}health\;center\;beween\;1\;to\;30\;min_{ij}\\ & +\beta_{25}health\;center\;between\;31\;to\;60\;min_{ij}\\ & +\beta_{26}health\;center\;between\;61\;to\;90\;min_{ij}\\ & +\beta_{27}health\;center\;between\;91\;to\;150\;min_{ij}\\ & +\beta_{28}not\;on\;electricity\;grid_{ij}+\beta_{29}\;on\;electricity\;grid_{ij}\\ & +\beta_{30}mean\;centered\;wealth\;scores_{ij} \end{array}$$

$$\beta_{0ij}=\beta_{0}+\upsilon_{0j}+\;\varepsilon_{0ij}$$

$$\beta_{30ij}=\beta_{30}+\upsilon_{30j}+\varepsilon_{30ij}$$

$$\left[\begin{array}{c} \upsilon_{0j}\\ \upsilon_{30j} \end{array}\right]\;∼\;N\left(0,\Omega_{u}\right):\Omega_{u}=\left[\begin{array}{cc} \sigma_{u0}^{2} & \\ \sigma_{u030} & \sigma_{u30}^{2} \end{array}\right]$$

$$\left[\begin{array}{c} \varepsilon_{0j}\\ \varepsilon_{30j} \end{array}\right]\;∼\;N\left(0,\Omega_{\varepsilon }\right):\Omega_{\varepsilon }=\left[\begin{array}{cc} \sigma_{\varepsilon 0}^{2} & \\ \sigma_{\varepsilon 030} & \sigma_{\varepsilon 30}^{2} \end{array}\right]$$

In this two-level regression model, ${Cov}_{ij}$ is the vaccination coverage or status of the mother *i*’s child in the neighborhood *j*. $\upsilon_{0j},\upsilon_{30j}$ are variances in measles vaccination status for variable $x_{0},\;x_{30}$ in the neighborhood *j*. $u_{0j},u_{30j}$ are random effects at the neighborhood level. $\varepsilon_{0j},\;\varepsilon_{30j}$ are the random effects at the mother level. $\varepsilon_{ij},\;\varepsilon_{30j}$ represent the differentials in measles vaccination status for variables $x_{0},\;x_{30}$ for mother *i* in district *j.* When the individual-level variables are mean-centered, between-mother effects, regardless of neighborhood, are detected for wealth scores.

## 3. Results

### 3.1. Study Population Characteristics

A total of 460 mothers were recruited for this study. Their children were presumed to have finished routine vaccination before turning one year old. Children aged 12–23 months were included; of 460, 15 children over or under the age criteria were excluded from the data analysis.

Table 1 shows the individual- and neighborhood-level descriptive characteristics of the mothers, according to their children’s measles vaccination status. Regarding individual-level factors, a little over a third (33.7%, 150 mothers) had never attended school. Approximately 40% of mothers (182 mothers) had attended both primary and secondary school. Of these primary- and secondary-educated mothers, 70.3% had children vaccinated against measles 1. Nearly nine out of ten mothers had given birth at a health center (398 mothers out of 445).

For the neighborhood-level factors, access time to the health center and access to electricity (in the household) were examined. Almost three-quarters (73.8%) of the mothers lived within 30 min of the health center. Three-quarters of the mothers surveyed had access to electricity. Of these, 219 mothers (65.2%) had vaccinated their children with the measles 1 vaccine.

### 3.2. Map of Measles 1 Vaccination Coverage by Neighborhood

**Figure 1:**
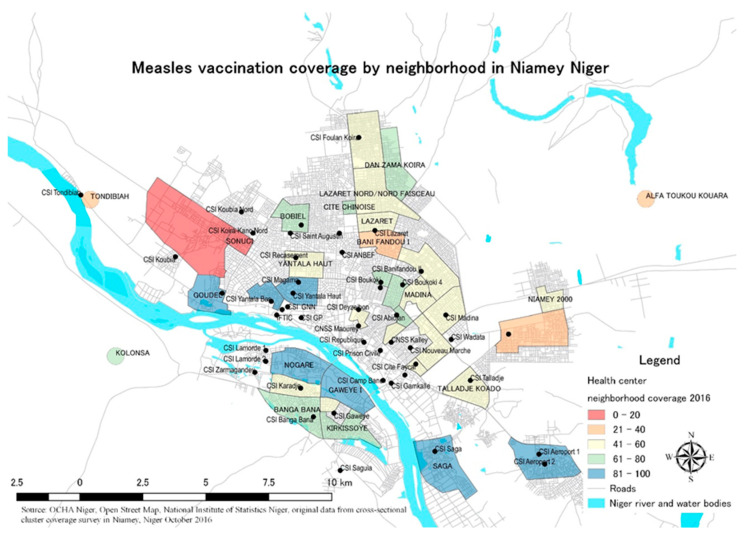
Measles 1 vaccination coverage (%) by neighborhood in Niamey, Niger. Source: base maps from OCHA Niger, Open Street Map, and National Institute of Statistics Niger. Measles 1 vaccination coverage data from Kondo Kunieda et al. (2021).

Figure 1 shows a map of measles 1 vaccination coverage by neighborhood. The black dots in the map represent local health centers. The access time to local health centers varied among mothers. For the least-vaccinated neighborhoods of the Koubia Nord and Kolonsa neighborhoods, nine mothers had to travel for 60 min to reach the health center. One mother in Koubia Nord had to travel 70 min and another mother travelled 90 min to reach the health center. In Bani Fandou I, with 40.0% measles 1 coverage, access time to the health center was 10 min for nine mothers and 20 min for one mother. For Sari Koubou et Kobontafa mothers, two lived within 30 min of the health center, two within 45 min, three within 60 min, one said it took 120 min, and two replied that it took them 150 min to reach the health center. For Nogare neighborhood mothers, access time to the health center was between 8 min and 40 min, with 50.0% living 15 min away from the health center. For the outlying villages without a health center, 7 out of 10 mothers who took their children to receive measles vaccination had to travel 120 min.

None of the respondents in the Kolonsa neighborhood had access to electricity. Four out of seven respondents (57.1%) in the Koubia Nord neighborhood did not have electricity. Of the 10 respondents from Bani Fandou I, 10 (100.0%) had access to electricity. Seven out of ten respondents (70.0%) in the Sari Koubou et Kobontafa neighborhood had access to electricity. In the Nogare neighborhood, eight out of ten respondents (80.0%) had access to electricity.

### 3.3. Multilevel Logistic Regression

When a null model (model 0) was run with measles 1 vaccination coverage as the dependent variable, some neighborhood-level variance (0.29) was detected. Therefore, four multilevel models were constructed and tested. The results are presented in Table 2. Measures of association were reported as odds ratios (ORs) with 95% confidence intervals (CIs). Variations were reported using the variance partition coefficient (VPC), intraclass correlation (ICC), proportional change in cluster variation (PCV), and median odds ratio (MOR) [35,36]. VPC represents the proportion of all observed individual variations in measles 1 vaccination attributable to neighborhood-level variation. In a simple multilevel structure of mothers nested within the neighborhood, VPC is equivalent to ICC. ICC is the percentage of total variance in the odds of measles 1 vaccination at the neighborhood level. MOR is the probability of measles 1 vaccination attributed to the neighborhood context. The MOR is a measure of heterogeneity between neighborhoods; in general, the larger the MOR, the larger the variance. However, the MOR value should be interpreted with respect to the VPC (ICC). In this case, even a seemingly large MOR would be considered low if the VPC (ICC) is small. PCV, or the proportional change in variance, quantifies neighborhood-level variation. A lower Akaike information criterion (AIC) indicates a better model fit.

Model 0 identified a 1.71 odds ratio of a child’s measles 1 vaccination in a certain neighborhood when all the independent variables were equal to zero. VPC was 0.08, which translates to 8% mother-level differences in the underlying propensity for her child to be measles 1 vaccinated. This 8% variation is due to between-neighborhood systematic differences, while the remaining 92% is due to within-mother systematic differences. The ICC was 8.1%, indicating a relatively small between-group variance and a larger within-group variance. If two mothers were randomly selected from the same neighborhood, the ICC would be the correlation between the two mothers in measles 1 vaccination.

Model 1 focused on individual-level factors, such as mother’s education level, mother’s age, mother tongue, and husband’s job, where the mother had given birth and whether she discussed vaccination with friends. In this model, primary and secondary-educated mothers were more likely to vaccinate their children against measles 1 (aOR 1.92, 95% CI 1.14–3.22). In addition, model 1 found that 13.8% of the neighborhood level differences could be explained by individual-level factors.

Model 2 included neighborhood-level variables, which were access time to the health center and access to electricity or whether the mother lived in a neighborhood on the electricity grid. The between-neighborhood variance was 7.1%. PCV was 13.8%. The MOR was the smallest (MOR 1.61) among the four models, indicating the smallest variance among neighborhoods. Model 2 was the best-fit model, with an AIC of 517.21.

The full multilevel model 3 was the second-best fit model, with an AIC of 530.93 among the four multilevel models. The MOR was 1.68, and the VPC and ICC were both 8%. Neighborhood-level PCV was −3.5%. In this model, primary and secondary-educated mothers were more likely to vaccinate their children against measles 1 (aOR 1.97, 95% CI 1.11–3.51).

The results of the null model, model 1, model 2, and model 3 demonstrate, through the VPC and MOR, a small magnitude neighborhood-level effect, with little variation between neighborhoods.

The neighborhood context was relatively homogeneous in terms of electricity and wealth. However, access time to health centers varied widely among mothers. Mothers who lived more than 30 min away from the health center were less likely to vaccinate their children against measles 1. Although not a significant association, mothers who lived furthest away from the health center (between 91 to 150 min) were twice as likely than those who lived close by to vaccinate against measles 1. Of the ten mothers who lived furthest away but had vaccinated their children against measles 1, seven were between the ages of 30 and 39 years (data not shown).

## 4. Discussion

In this study, the individual-level factor of primary and secondary educated mothers indicated a higher likelihood of mothers to vaccinate their children against measles 1 (aOR 1.97, 95% CI 1.11–3.51). At the neighborhood-level, no factors associated with measles 1 vaccination coverage were identified. The variance partition coefficient (VPC) of 0.08 meant that only a small proportion of all the observed individual variations in measles 1 vaccination were attributable to neighborhood-level variation. 

### 4.1. Individual-Level Factors Associated with Measles 1 Vaccination

This study identified mother’s primary and secondary education as a factor for her child’s measles 1 vaccination. In reality, Nigerien girls’ access to primary and secondary education has improved over the last decade [37]. The World Bank estimates that lower secondary education completion rates for urban girls improved from 17.7% (2006) to 19.7% (2016) in particularly urban areas. Upper secondary education completion rates for urban girls has risen from 1.9% (2006) to 4.9% (2012) [26]. The same World Bank data on measles coverage for children aged 12–23 months show an improvement from 37% in 2000, to 53% in 2006 and 75% in 2012 [26]. Therefore, it is possible that measles 1 vaccination will improve as more mothers complete their primary and secondary education.

Examining how decisions are made by low-literate mothers may also be necessary. A qualitative study conducted in Australia found that participants with less education tended to consent to the option recommended by the doctor, but not necessarily agreeing with the recommendation [38]. These low-literate participants were frequently consulted and played a key role in their relatives’ and friends’ decisions. They also admitted that their health worker’s interpersonal communication skills influenced them. A qualitative enquiry might be necessary to investigate whether this is also happening in Niamey’s low-literacy environment.

### 4.2. Neighborhood-Level Factors Associated with Measles 1 Vaccination

At the neighborhood-level, contrary to a multi- and national-level Ethiopian study, no factors associated with measles 1 vaccination were identified through the multilevel analysis [39]. This may have been because the enumeration areas and neighborhoods were too small to detect differences. Another issue might have been the focus on infrastructure-related, constructed neighborhood variables of access time to health centers, access to electricity, and mean-centered individual wealth scores. These variables may not have been optimal as neighborhood-level factors in determining measles vaccine uptake.

According to local health workers, mothers are not restricted to their nearest health center. Mothers who live on the north side of the river Niger can access any health center with which they are familiar and that meets their expectations in terms of environment and worker attitudes. This access multiplicity may have led to skewed neighborhood-level results.

Another supply-side constraint, especially for the peripheral, almost rural health centers in the capital, would be the 10-dose vials for BCG and measles vaccination [40,41]. Health workers would be reluctant to open the vial if there were fewer than 10 infants (between the ages of 9 and 12 months for measles vaccination) on that day. Vaccination services are not routinely provided, and mothers are forced to travel to other health centers that provide routine vaccination services.

Niamey is a rapidly growing city, with little distinction between rich, poor, or slum neighborhoods [30,42]. If a neighborhood does not have access to the electricity grid, this is not necessarily because it is a poor neighborhood, but because this neighborhood is new and there is still no electricity grid [43]. If the households do not have electricity, the health center will probably also not have electricity, or a refrigerator to store vaccines [44]. In this case, the unelectrified health center relies on the nearest health center to keep vaccines and supply them through cold carriers.

### 4.3. Actionable Factors for Measles Vaccination Coverage

If individual-level factors are of relative importance, more research is required to identify the unidentified, highest-impact individual-level factors. At the same time, if neighborhoods do not account for a large difference in results, a multilevel analysis might not always be necessary. The multilevel analysis relies on the mean to calculate the differences in intercepts and slopes between individuals and neighborhoods. Instead of erasing the differences between individuals using the mean, more individual differentiation factors are necessary for a better understanding of the problem [45]. Factors should consider individual characteristics, such as habits, individual motivation, and the ability to access vaccination services, as well as direct prompts that trigger such motivation and ability [46,47]. The ability to access vaccination services also includes child health or vaccination-specific decision-making power within the household, as a recent study identified that the medium decision-making power of the mother is associated with complete vaccination of her child [48].

One behavior that might be interesting to examine further is “discussion on vaccination.” In this study, questions on “discussion on vaccination” with family and friends were asked at the individual-level. However, at an aggregated, neighborhood-level, properly guided “discussions on vaccination” programs could lead to the creation of a positive social norm regarding vaccination. Mothers might receive information on vaccination, or in the Indian case, through an information hub and SMS reminders [13]. These would work as prompts for discussing vaccination among family and friends. By participating in a discussion on vaccination, mothers may be motivated to access vaccination. More specifically, discussions may enable mothers to overcome their inabilities to access vaccination together and make sure that, together, they coordinate their visit and a multi-dose measles vaccine vial can be opened.

Another study on rubella seroprevalence in Niger, using approximately 40% of the suspected measles cases of surveillance data, found a national prevalence of 7%. The sero-prevalence in the Maradi region was four times higher, in the Tahoua region, it was 2.5 times higher and in the Zinder region, it was 2 times higher. There were no urban or rural differences, and the majority of cases were reported from January to April, with a peak in April [49]. Such scientific information could be diffused to create discussions and readiness to vaccinate when measles and rubella campaigns are implemented.

In a previous study on access to health facilities in Niger, 67% of children living farther than one hour from the health center, compared to the 44% of those living within one hour, were completely vaccinated by the time they had turned one year old [32]. There might be a difference in full vaccination and measles vaccination, as this study found that an access time between 61 and 90 min seems to be a deterrent to vaccination. Another measles uptake study identified a similar trend that the longer the travel time to the health facility, the less measles uptake [50]. Although the results were insignificant, ensuring that planned outreach and mobile vaccination activities are realized is critical in improving individual- and neighborhood-level vaccination coverage. Figure 1 shows that vaccination coverage is low (red) in neighborhoods with a just-opened health center far from the majority of its residents. However, the outlying rural village Alpha Toukou Kouara did not have a health center, but had higher vaccination coverage than this urban neighborhood. Seven out of ten mothers had travelled for 120 min to get their children’s measles vaccination. These examples of positive deviance show the importance of motivation to overcome distance.

### 4.4. Limitations

The present study has several limitations. First, information on contextual factors, such as living and housing environments, was not collected. Such information would have provided more information related to measles infection risk factors, such as whether the families lived in aired or unaired, open or closed, close-contact rooms. Overall, sampling and collecting data for vaccine uptake-related neighborhood variables is a limitation of this study. Second, as the questionnaire was based on the traditional DHS questionnaire and coverage survey questionnaire, data were not collected on individual influences, such as religious beliefs, media access, cultural drivers, and decision-making power, which might have uncovered more insight into the individual-level factors associated with measles vaccine uptake. Third, not all Niamey’s enumeration areas were surveyed, preventing a full-scale spatial analysis. Fourth, despite an extensive search for studies on health promotion and vaccine decision-making among low-literacy African populations, none were directly relevant. Studies were either conducted outside Africa or not specifically on low-literacy populations. Finally, the cross-sectional study design limits the determination of cause-and-effect relationships.

## 5. Conclusions

At the individual-level, primary and secondary-educated mothers were more likely to vaccinate their children against measles 1. At the neighborhood-level, no factors were identified. Therefore, a strengthened focus on equity-based, individual factors is recommended, including individual motivation, prompts and ability to access vaccination services.

## Figures and Tables

**Table 1:** Study population characteristics.

Variable	Total		Child Vaccinated Against Measles 1		Child Not Vaccinated Against Measles 1		
	*n*	(%)	*n*	(%)	*n*	(%)	*p*-Value
**Maternal Education Level**							0.037
No education	150	(33.7)	84	(56.0)	66	(44.0)	
Basic literacy	87	(19.6)	50	(57.5)	37	(42.5)	
Primary-secondary education	182	(40.9)	128	(70.3)	54	(29.7)	
Secondary plus education	26	(5.8)	17	(65.4)	9	(34.6)	
**Mother’s Age**							0.433
15 to 19 years old	32	(7.2)	17	(53.1)	15	(46.9)	
20 to 24 years old	103	(23.3)	69	(66.0)	35	(34.0)	
25 to 29 years old	132	(29.8)	86	(65.2)	46	(34.9)	
30 to 34 years old	80	(18.1)	44	(55.0)	36	(45.0)	
35 to 39 years old	73	(16.5)	48	(65.8)	25	(34.3)	
40 years old or more	23	(5.2)	16	(30.4)	7	(69.6)	
**Mother Tongue**							0.912
Hausa	143	(32.1)	87	(60.8)	56	(39.2)	
Zarma	240	(53.9)	154	(64.2)	86	(35.8)	
Fulani	35	(7.9)	22	(62.9)	13	(37.1)	
Tamachek	8	(1.8)	4	(50.0)	4	(50.0)	
Others	19	(4.3)	12	(63.2)	7	(36.8)	
**Husband’s Job**							0.464
Unemployed	19	(4.3)	13	(68.4)	6	(31.6)	
Informal work	194	(43.6)	119	(61.3)	75	(38.7)	
Formal employment	147	(33.0)	88	(59.9)	59	(40.1)	
Public servant	85	(19.1)	59	(69.4)	26	(30.6)	
**Gave Birth**							0.017
At home	47	(10.6)	22	(46.8)	25	(53.2)	
At health center	398	(89.4)	257	(64.6)	141	(35.4)	
**Mother Discussed Vaccination with Friends**							0.311
No discussion	163	(37.7)	100	(61.4)	63	(38.7)	
Had discussed	269	(62.3)	178	(66.2)	91	(33.8)	
**Time to (Access) Health Center**							0.033
1 to 30 min	302	(73.8)	204	(67.6)	98	(32.5)	
31 to 60 min	84	(20.5)	49	(58.3)	35	(41.7)	
61 to 90 min	10	(2.4)	3	(30.0)	7	(70.0)	
91 to 150 min	13	(3.2)	10	(76.9)	3	(23.1)	
**Access to Electricity**							0.076
No	101	(23.1)	56	(55.5)	45	(44.6)	
Yes	336	(76.9)	219	(65.2)	117	(34.8)	
**Mean Centered Wealth Scores**							0.092
Total	436		275	(63.1)	161	(36.9)	

*p*-value *: significant at the 5% level.

**Table 2:** Factors of measles 1 vaccination from multilevel logistic regression models.

Variables	Model 0 (Empty Model)		Model 1 (Only Individual Level Variables)		Model 2 (Only Neighborhood-Level Variables)		Model 3 (Multilevel)	
	*n* = 445		*n* = 431		*n* = 401		*n* = 400	
	OR	(95% CI)	OR	(95% CI)	OR	(95% CI)	OR	(95% CI)
**Maternal Education**								
No education (ref)								
Basic literacy			1.05	(0.57–1.95)			0.94	(0.49–1.82)
Primary-secondary education			1.92 *	(1.14–3.22)			1.97 *	(1.11–3.51)
Secondary plus education			1.75	(0.60–5.13)			1.53	(0.48–4.83)
**Mother’s Age**								
15 to 19 years old (ref)								
20 to 24 years old			1.72	(0.73–4.05)			1.40	(0.56–3.53)
25 to 29 years old			1.81	(0.78–4.20)			1.62	(0.65–4.04)
30 to 34 years old			1.27	(0.53–3.06)			1.01	(0.39–2.60)
35 to 39 years old			2.01	(0.80–5.06)			1.87	(0.70–5.00)
40 years old or more			3.01	(0.82–10.98)			3.91	(0.90–16.92)
**Mother Tongue**								
Hausa (ref)								
Zarma			1.05	(0.64–1.72)			1.17	(0.69–1.98)
Fulani			0.98	(0.38–2.52)			1.35	(0.47–3.85)
Tamachek			0.84	(0.15–4.62)			1.27	(0.21–7.50)
Others			0.93	(0.31–2.73)			0.93	(0.29–3.02)
**Husband’s Job**								
Unemployed (ref)								
Informal work			0.51	(0.16–1.66)			0.47	(0.13–1.64)
Formal employment			0.51	(0.15–1.68)			0.54	(0.15–1.95)
Public servant			0.63	(0.18–2.24)			0.51	(0.13–1.99)
**Gave Birth**								
At home (ref)			1.83	(0.85–3.94)				
At health center							2.04	(0.84–4.94)
**Mother Discussed Vaccination with Friends**								
No discussion (ref)								
Had discussed			1.25	(0.75–2.07)			1.09	(0.63–1.87)
**Time to (Access) Health Center**								
1 to 30 min (ref)								
31 to 60 min					0.68	(0.39–1.18)	0.71	(0.39–1.28)
61 to 90 min					0.23	(0.05–1.02)	0.23	(0.04–1.26)
91 to 150 min					2.35	(0.51–10.76)	4.12	(0.77–22.03)
**Access to Electricity**								
No (ref)								
Yes					1.06	(0.50–2.26)	0.85	(0.38–1.92)
**Mean Centered Wealth Scores**					1.11	(0.92–1.34)	1.11	(0.90–1.36)
Intercept (SE)	1.71	(0.23)	0.77	(0.63)	2.02	(0.69)	1.06	(1.00)
**Random Effects**								
Neighborhood variance (SE)	0.29	(0.19)	0.25	(0.20)	0.25	(0.20)	0.30	(0.23)
**VPC**	0.08		0.07		0.07		0.08	
**PCV (%)**	-		13.8%		13.8%		−3.5%	
**Interclass Correlation (ICC, %)**	8.1%		7.1%		7.1%		8.3%	
**Median Odds Ratio (MOR)**	1.67		1.61		1.61		1.68	
**Log Pseudolikelihood**	−291.61		−268.48		−251.60		−241.47	
**AIC**	587.23		574.96		517.21		530.93	

## Data Availability

The dataset and questionnaire are available from Harvard Dataverse (DOI: https://doi.org/10.7910/DVN/XQ2NSO, accessed on 10 June 2022).

## References

[1] Black R.E., Laxminarayan R., Temmerman M., Walker N. (2016). Reproductive, Maternal, Newborn, and Child Health Disease Control Priorities.

[2] WHO (2018). Explorations of Inequality: Childhood Immunization.

[3] Kazungu J.S., Adetifa I.M.O. (2017). Crude childhood vaccination coverage in West Africa: Trends and predictors of completeness. Wellcome Open Res..

[4] Russo G., Miglietta A., Pezzotti P., Biguioh R.M., Bouting Mayaka G., Sobze M.S., Stefanelli P., Vullo V., Rezza G. (2015). Vaccine coverage and determinants of incomplete vaccination in children aged 12–23 months in Dschang, West Region, Cameroon: A cross-sectional survey during a polio outbreak. BMC Public Health.

[5] Schoeps A., Ouédraogo N., Kagoné M., Sié A., Müller O., Becher H. (2013). Socio-demographic determinants of timely adherence to BCG, Penta3, measles, and complete vaccination schedule in Burkina Faso. Vaccine.

[6] Kunieda M.K., Manzo M.L., Shibanuma A., Jimba M. (2021). Rapidly modifiable factors associated with full vaccination status among children in Niamey, Niger: A cross-sectional, random cluster household survey. PLoS ONE.

[7] Gage A.J., Sommerfelt A.E., Piani A.L. (1997). Household structure and childhood immunization in Niger and Nigeria. Demography.

[8] Bangura J.B., Xiao S., Qiu D., Ouyang F., Chen L. (2020). Barriers to childhood immunization in sub-Saharan Africa: A systematic review. BMC Public Health.

[9] Douba A., Aka L.B.N., Yao G.H.A., Zengbé-Acray P., Akani B.C. (2015). Sociodemographic factors associated with incomplete immunization of children aged 12 to 59 months in six West African countries. Sante Publique.

[10] Uthman O.A., Sambala E.Z., Adamu A.A., Ndwandwe D., Wiyeh A.B., Olukade T., Bishwajit G., Yaya S., Okwo-Bele J.-M., Wiysonge C.S. (2018). Does it really matter where you live? A multilevel analysis of factors associated with missed opportunities for vaccination in sub-Saharan Africa. Hum. Vaccines Immunother..

[11] Tesema G.A., Tessema Z.T., Tamirat K.S., Teshale A.B. (2020). Complete basic childhood vaccination and associated factors among children aged 12–23 months in East Africa: A multilevel analysis of recent demographic and health surveys. BMC Public Health.

[12] Oyo-Ita A., Wiysonge C., Oringanje C., E Nwachukwu C., Oduwole O., Meremikwu M.M. (2016). Interventions for improving coverage of childhood immunisation in low- and middle-income countries. Cochrane Database Syst. Rev..

[13] Banerjee A., Chandrasekhar A., Dalpath S., Duflo E., Floretta J., Jackson M., Kannan H., Loza F., Sankar A., Schrimpf A. (2021). Selecting the Most Effective Nudge: Evidence from a Large-Scale Experiment on Immunization.

[14] Shetty P. (2010). Nutrition, Immunity and Infection.

[15] Bartlett S. (2016). Water, sanitation and urban children: The need to go beyond “improved” provision. Environ. Urban..

[16] Alirol E., Getaz L., Stoll B., Chappuis F., Loutan L. (2011). Urbanisation and infectious diseases in a globalised world. Lancet Infect. Dis..

[17] Grais R., Ferrari M., DuBray C., Bjørnstad O., Grenfell B., Djibo A., Fermon F., Guerin P. (2006). Estimating transmission intensity for a measles epidemic in Niamey, Niger: Lessons for intervention. Trans. R. Soc. Trop. Med. Hyg..

[18] Besada D., Kerber K., Leon N., Sanders D., Daviaud E., Rohde S., Rohde J., Van Damme W., Kinney M., Manda S. (2016). Niger’s Child Survival Success, Contributing Factors and Challenges to Sustainability: A Retrospective Analysis. PLoS ONE.

[19] Cockcroft A., Andersson N., Omer K., Ansari N.M., Khan A., Chaudhry U.U., Ansari U. (2009). One size does not fit all: Local determinants of measles vaccination in four districts of Pakistan. BMC Int. Health Hum. Rights.

[20] Sissoko D., Trottier H., Malvy D., Johri M. (2014). The Influence of Compositional and Contextual Factors on Non-Receipt of Basic Vaccines among Children of 12–23 Month Old in India: A Multilevel Analysis. PLoS ONE.

[21] Halonen J.I., Kivimäki M., Pentti J., Kawachi I., Virtanen M., Martikainen P., Subramanian S.V., Vahtera J. (2012). Quantifying Neighbourhood Socioeconomic Effects in Clustering of Behaviour-Related Risk Factors: A Multilevel Analysis. PLoS ONE.

[22] Kinfe Y., Gebre H., Bekele A. (2019). Factors associated with full immunization of children 12–23 months of age in Ethiopia: A multilevel analysis using 2016 Ethiopia Demographic and Health Survey. PLoS ONE.

[23] Antai D. (2010). Migration and child immunization in Nigeria: Individual- and community-level contexts. BMC Public Health.

[24] Antai D. (2009). Inequitable childhood immunization uptake in Nigeria: A multilevel analysis of individual and contextual determinants. BMC Infect. Dis..

[25] Wiysonge C.S., Uthman O.A., Ndumbe P.M., Hussey G.D. (2012). Individual and Contextual Factors Associated with Low Childhood Immunisation Coverage in Sub-Saharan Africa: A Multilevel Analysis. PLoS ONE.

[26] World Bank (2020). Niger Education Statistics. https://data.worldbank.org/country/niger.

[27] WHO, UNICEF Measles vaccination coverage (for Niger by year). https://immunizationdata.who.int/pages/coverage/mcv.html?CODE=NER&ANTIGEN=&YEAR=.

[28] INS (2013). Présentation des résultats préliminaires du quatrième (4ème) Recensement Général de la Population et de l’Habitat (RGP/H) 2012.

[29] UN Department of Economic and Social Affairs, Statistics Division (2016). World Statistics Pocketbook 2016 edition World Statistics Pocketbook Series V.

[30] Rossi J.-P., Dobigny G. (2019). Urban Landscape Structure of a Fast-Growing African City: The Case of Niamey (Niger). Urban Sci..

[31] ICF, INS (2012). Enquête Démographique et de Santé et à Indicateurs Multiples du Niger 2012.

[32] Blanford J.I., Kumar S., Luo W., MacEachren A.M. (2012). It’s a long, long walk: Accessibility to hospitals, maternity and integrated health centers in Niger. Int. J. Health Geogr..

[33] Sommet N., Morselli D. (2017). Keep Calm and Learn Multilevel Logistic Modeling: A Simplified Three-Step Procedure Using Stata, R, Mplus, and SPSS. Int. Rev. Soc. Psychol..

[34] Subramanian S., Jones K. (2017). Multilevel Statistical Models: Concepts and Applications, 2017–2018 ed..

[35] Austin P.C., Merlo J. (2017). Intermediate and advanced topics in multilevel logistic regression analysis. Stat. Med..

[36] Merlo J., Chaix B., Ohlsson H., Beckman A., Johnell K., Hjerpe P., Råstam L., Larsen K. (2006). A brief conceptual tutorial of multilevel analysis in social epidemiology: Using measures of clustering in multilevel logistic regression to investigate contextual phenomena. J. Epidemiol. Community Health.

[37] UNESCO (2021). Niger: Participation in Education. http://uis.unesco.org/en/country/ne.

[38] Smith S.K., Dixon A., Trevena L., Nutbeam D., McCaffery K.J. (2009). Exploring patient involvement in healthcare decision making across different education and functional health literacy groups. Soc. Sci. Med..

[39] Geremew T.T., Gezie L.D., Abejie A.N. (2019). Geographical variation and associated factors of childhood measles vaccination in Ethiopia: A spatial and multilevel analysis. BMC Public Health.

[40] Byberg S., Fisker A.B., Rodrigues A., Balde I., Enemark U., Aaby P., Benn C.S., Griffiths U.K. (2016). Household experience and costs of seeking measles vaccination in rural Guinea-Bissau. Trop. Med. Int. Health.

[41] Assi T.M., Brown S.T., Djibo A., Norman B.A., Rajgopal J., Welling J.S., Chen S.I., Bailey R.R., Kone S., Kenea H. (2011). Impact of changing the measles vaccine vial size on Niger’s vaccine supply chain: A computational model. BMC Public Health.

[42] HABITAT (2007). Profil Urbain National du Niger.

[43] Abdourazack N.A. (2017). Urbanisation et Précarité de L’énergie Électrique dans les Grandes Villes d’Afrique de l’Ouest: L’exemple de Niamey au Niger (Analyse Bibliographique), in Faculté des Lettres et Sciences Humaines Département de Géographie.

[44] Chen Y.J., Chindarkar N., Xiao Y. (2019). Effect of reliable electricity on health facilities, health information, and child and maternal health services utilization: Evidence from rural Gujarat, India. J. Health Popul. Nutr..

[45] Rose T. (2016). The End of Average: How We Succeed in a World That Values Sameness.

[46] Agha S., Tollefson D., Paul S., Green D., Babigumira J.B. (2019). Use of the Fogg Behavior Model to Assess the Impact of a Social Marketing Campaign on Condom Use in Pakistan. J. Health Commun..

[47] Fogg B. (2019). Tiny Habits: The Small Changes That Change Everything.

[48] Seidu A.-A., Ahinkorah B.O., Ameyaw E.K., Budu E., Yaya S. (2021). Women empowerment indicators and uptake of child health services in sub-Saharan Africa: A multilevel analysis using cross-sectional data from 26 countries. J. Public Health.

[49] Moumouni A., Doingalé H., Mahamadou D., Attoh T., Tiembré I. (2021). Séroprévalence de la Rubéole au Niger de 2005 à 2019: Estimations Issues du Système de Surveillance Épidémiologique de la Rougeole. St. Publique.

[50] Aheto J.M.K., Pannell O., Dotse-Gborgbortsi W., Trimner M.K., Tatem A.J., Rhoda D.A., Cutts F.T., Utazi C.E. (2022). Multilevel analysis of predictors of multiple indicators of childhood vaccination in Nigeria. PLoS ONE.

